# Partially orthogonal resonators for magnetic resonance imaging

**DOI:** 10.1038/srep42347

**Published:** 2017-02-10

**Authors:** Jorge Chacon-Caldera, Matthias Malzacher, Lothar R. Schad

**Affiliations:** 1 Computer Assisted Clinical Medicine, Medical Faculty Mannheim, Heidelberg University, Mannheim, Germany

## Abstract

Resonators for signal reception in magnetic resonance are traditionally planar to restrict coil material and avoid coil losses. Here, we present a novel concept to model resonators partially in a plane with maximum sensitivity to the magnetic resonance signal and partially in an orthogonal plane with reduced signal sensitivity. Thus, properties of individual elements in coil arrays can be modified to optimize physical planar space and increase the sensitivity of the overall array. A particular case of the concept is implemented to decrease H-field destructive interferences in planar concentric in-phase arrays. An increase in signal to noise ratio of approximately 20% was achieved with two resonators placed over approximately the same planar area compared to common approaches at a target depth of 10 cm at 3 Tesla. Improved parallel imaging performance of this configuration is also demonstrated. The concept can be further used to increase coil density.

Signal-to-noise ratio (SNR) is a key factor in magnetic resonance imaging (MRI). SNR can limit the minimum scanning times and applications, the maximum resolution, and could also determine the diagnostic quality of the images. The maximum SNR is limited by the MRI system, the measurement setup and the signal reception elements^1^,^2^. For a given setup and system, increasing SNR at a region of interest (ROI) is increasingly challenging as the depth of the ROI increases. The challenge is present even in innovative approaches such as the use of metamaterials which focus the sensitivity field but obtain maximum SNR gains from objects near the coil i.e. superficial areas of the body^3^,^4^. Therefore, the optimization of reception elements for MRI is necessary to maximize SNR especially for deep tissues in the body.

Historically, one of the main priorities in the design of reception elements for MRI has been to fit the elements to the imaged volume tightly. In typical approaches, since the early 90 s, this is done using small planar or approximately planar elements that are located around the surface of the sample regardless of the application^5^,^6^,^7^,^8^. Their purpose is to maximize the detected signal arising from the volume while the noise arising from the coil resistance is minimized. Practically, this is achieved by making the whole elements sensitive to the signal and minimizing both the coil conductor material and lumped elements to reduce losses^9^. This has been thus far considered ideal since the effective resistance of the MRI experiment includes the coil resistance (R_coil_) as:


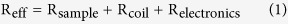


The irrefutability of the argument that R_coil_ introduces losses has longstanding restricted the design of the resonators. However, in clinical MRI applications, the resistance is largely dominated by the sample^10^.

In this work, we rely on the latter fact and introduce the novel concept of partially orthogonal resonators in which each coil is partly sensitive and partially insensitive to the MRI signal. The insensitive part is formed by an added area orthogonal to the plane with maximum sensitivity. This is done to modify the interactions between elements in an array and add flexibility to its design. Using this concept, it is possible to overcome limitations in plane arrays and improve their overall performance. A basic example is the arrangement of two single loop coils decoupled by overlap. The overlap area, and therefore, the total planar area occupied by two partially orthogonal single loop coils can be modified by changing the height of the orthogonal area. This adds flexibility to the design of arrays in comparison to the traditional planar single loops with overlap where the overlap area and the total planar area are fully determined by the size of the coils (see Fig. 1).

One of the possibilities using the partially orthogonal resonators is to increase coil density in arrays using overlap decoupling. Another possibility is to use a special case of the partially orthogonal resonators to place two coils with geometrical decoupling over virtually the same area. Thus, each coil is sensitive to virtually the same volume which can be achieved using orthogonal coils.

An example is the butterfly or figure-8 coil which shows inductive decoupling when placed concentrically with a single loop coil^11^. This has been used in previous studies as a quadrature pair to detect the full complex magnetization^12^,^13^,^14^. Although quadrature detection is desired to increase performance^15^, further SNR gain can be obtained by increasing in-phase signal detection prior to adding an element for quadrature detection. In-phase concentric coils subdivided into “lobes” generate H-fields in opposite direction for different lobes. This creates intrinsic H-field interferences with neighbor coils^16^ which affects the **B**_1_ field of the array and the sensitivity from the point-of-view of reciprocity. In this work, we take this case to improve the performance of planar concentric arrays by redirecting the areas where the H-fields of the two coils oppose to a plane with reduced sensitivity to the MR signal. The aim is to reduce H-field destructive interferences when the coils are in-phase while focusing the sensitivity to a target planar area. Our approach is to use only the part of the array where the two coils yield a constructive interference of the H-field for signal detection. The part of the array with destructive interference can be placed in an orthogonal plane where the influence of the interference does not affect the signal reception (see Fig. 2). This is done ultimately to address the challenging purpose of increasing SNR at a target depth. SNR at other target depths can be optimized by changing the size of the coils. The proposed array is compared to common approaches.

## Results

### Simulations

In the comparison of the partially orthogonal coil pair and the planar array consisting of a butterfly and a single loop coil, a reduction in H-field disturbances was observed using the absolute and vector H-field plots in the simulations. The vector plot of the H-fields showed higher directivity of the field towards the scanned volume (see Fig. 3). In terms of decoupling, for these two arrays, the scattering parameters used to assess it showed a lower absolute value of the forward voltage gain (S21) for the partially orthogonal coil array.

In the performance comparison against planar approaches, a higher sensitivity, estimated using the figure of merit (|**B**_1_^−^|_norm_), was yielded by the proposed partially orthogonal array. This comparison included the reference single loop, and the two single loops with overlap. Each one of the two arrays covered approximately the same planar area as the single loop. The maximum |**B**_1_^−^|_norm_ values for the planar arrays at a depth of 10 cm inside the phantom were: 355 nT for the single loop, 351 nT for the two single loops with overlap, and 338 nT for the concentric butterfly and single loop in-phase. The proposed array yielded |**B**_1_^−^|_norm_ = 382 nT.

E-fields were analyzed for the measured coil and arrays i.e. single loop, two-loo and partially orthogonal array. Here an increase in the E-field magnitudes was found, particularly outside the phantom when using the partially orthogonal coil. Inside the phantom, maximum E-field amplitudes were up to 76% higher compared to the planar two-loop array (transversal slice). However, mean E-field amplitudes were reduced in both slices tested compared to the planar array (see Fig. 4).

### Measurements

#### Phantom

Loaded Q factors (Q_Loaded_) of the elements in the proposed array were 1.6 and 1.5-fold greater (loop and butterfly) than the planar single loop. The unloaded-to-loaded ratios (Q_Unloaded_/Q_Loaded_) were greater than 4 in both partially orthogonal elements (see Table 1).

The geometric decoupling of the coil arrays measured in transmission was approximately −15 dB for the planar two single loops with overlap decoupling and approximately −10 dB for the partially orthogonal coil array. The MRI experiments showed an increase of SNR in the areas with greater distance to the planar area of the array when acquiring the data using the partially orthogonal coil pair in comparison to single loop and the two loops with overlap. This can also be seen in a profile taken through the center of the phantom. The SNR gains were obtained starting at a distance of 6 cm from the surface of the phantom and were observed until the far end of the phantom (see SNR Maps in Fig. 5). SNR gains were yielded in the whole measured volume by the proposed array in comparison to the single loop. Compared to the two single loops with overlap, gains were observed as the distance from the coil increased. At the target distance of 10 cm from the coil, an approximate 1.25-fold SNR increase was estimated when comparing the partially orthogonal coil pair to the single loop and an approximate 1.2-fold when compared to the two single loops array using biexponential fits (see SNR Ratios in Fig. 5). The noise correlations of the coils were: 20% for the two single loops with overlap and 14% for the partially orthogonal coil pair.

#### In vivo

The geometric decoupling due to the load of the sample was approximately −16 dB for the two single loops with overlap planar array and −13 dB for the proposed array. For the two single loops with overlap, the noise correlation was 31% and for the partially orthogonal coil pair was 13%. Images acquired using GRAPPA^17^ as parallel imaging strategy with a reduction factor of 2 showed decreased artifacts in the center of the brain by using the anterior-posterior direction for phase encoding. However, in the case of using left-right as a phase encoding direction, artifacts were more pronounced (see Fig. 6).

## Discussion

In this work, we introduced the novel concept of partially orthogonal coils. The concept differentiates itself from common approaches because parts of the coils are not used primarily for signal detection. This is contrary to the historical approach used thus far for signal detection, in which the entire coil is desired to be sensitive over a volume even if the element is partially orthogonal^18^. In other words, the partially orthogonal part of our concept is not used to extend the sensitive volume of the coil but rather to add flexibility to the design of coil arrays. On the other hand, all the elements of the partially orthogonal array have sensitivity to the signal, at least partially. This is also distinctive from previous approaches that employed entire elements virtually insensitive to the signal used to reduce E-fields^19^,^20^.

Based on this concept, we developed and tested an array of two partially orthogonal coils in-phase with sensitivity over virtually the same volume. Using this setup, the physical planar space placed over the sampled volume was optimized. At this point, it should be noticed that both coils in the proposed array contributed to the sensitivity. This is in contrast with previous designs using composite coils to achieve inductive decoupling, element to the surface of the sample^21^,^22^,^23^ or using small loops^24^ or solenoids^25^,^26^.

The initial comparison against planar elements was performed using a traditional planar concentric coil pair consisting of a single loop and a butterfly coil. In the simulations it showed destructive interferences which can also be seen as actively shielding the coils. The presented array reduced the H-field destructive interferences of the coils in the plane with maximum sensitivity to the MRI signal while using constructive interference of the coils in this plane. Using the same direction of the generated H-field, the |**B**_1_^−^|_norm_ was increased 13% using the simulations compared to the planar butterfly and single loop array in-phase. An increase of 8% in |**B**_1_^−^|_norm_ was also found in respect to the reference single loop and the common two-loop array with overlap. This was achieved despite the trade-off of increased mutual coupling between coils of the partially orthogonal coil pair in respect to the two loops with overlap in the simulations and the measurements. Although, a maximum S21 value of −13 dB was measured *in vivo* due to geometrical decoupling, the optimization of the design should include more efficient decoupling between the elements.

Increasing penetration depth is a well-known challenge in MRI^27^. This is because subdividing the planar area into smaller coils increases peripheral SNR but the gains decrease as the distance from the array increases^28^,^29^. Recently, this problem has been addressed by using travelling-wave^30^,^31^ and the combination of dipole antennas and single loop coils at 7 T^32^ in research. While travelling-wave has been found to create more homogeneous fields, local SNR was not increased. On the other hand, combinations in the form of stacks of loops and dipole antennas have provided signal to noise ratio increases but at 3 T the advantage of using dipoles instead of single loops is lost^33^. This is because the dimensions of the dipoles are constrained by the body and the bore size. Shortening the size of the dipole further reduces the efficiency of the dipoles. Therefore, at 3 T, current state-of-the-art approaches remain based on single loop coils placed laterally to each other decoupled using overlap^34^ methods. This is, as mentioned, done in the attempt to use the entire coil for MRI signal detection and thus, limit the coil noise.

Based on the novel concept of partially orthogonal resonators, the presented array showed an increase in SNR at the target depth. The gain was approximately 25% compared to the reference single loop optimized for the target depth and approximately 20% SNR compared two single loops with overlap covering the same area. An overall SNR gain in the volume was also obtained in comparison with the single loop as expected from the higher Q_Loaded_. This increase was due to the increased inductance and partial loading due to the orthogonal area. Compared to the two-loop planar array, a reduction in Q_Loaded_ suggests a lower sensitivity over the whole volume but SNR measurements demonstrated gains at the depth. This was also supported by our analysis of E-fields. Namely, the increase in peak amplitudes of E-fields in the phantom suggests an increase in dielectric losses which could reduce the SNR gains particularly at the surface. However, based on the reduced mean values in slices analyzed inside the phantom, it was estimated that the losses reduce dramatically as the distance between the coil and the object increase. The peak E-field amplitude was particularly high between the lobes of the butterfly in the partially orthogonal coil array proposed which presents room for optimization of the array.

In both cases, the gain was obtained despite the partial reduction of the sensitivity of the coil array due to the added orthogonal area. This was because the noise was sample dominated as indicated by the Q_Unloaded_/Q_loaded_ ratios greater than 4 yielded by the individual elements (see Supplementary Information). The proposed method presented a clear advantage to traditional methods considering the difficulties to enhance SNR at this depth. Increasing SNR in a target ROI (e.g. a voxel) is also a priority for spectroscopy. Given that RF coils can be applied for imaging as well as for spectroscopy^35^, the SNR increase of the proposed array could also be beneficial there. Considerations for the application of the array to spectroscopy include the dependency of coupling due to the resonance frequency (determined by the field and the nuclei) and the R_sample_/R_coil_ ratio. The enhancement in B-field amplitude could also be useful in transmission and transceiver modes.

SNR in the periphery was reduced compared to common approaches. Although achieving SNR at the periphery was not an aim in this study, this can be compensated by using arrays of the proposed pair.

Using *in vivo* brain scans, parallel imaging performance was assessed. For the chosen strategy (GRAPPA), an advantage was seen when using the anterior-posterior direction to encode the phase. A drawback was seen in the left-right direction for phase encoding. However, this drawback can be overcome by placing coils adjacent to the proposed array.

Building an array of the partially orthogonal coil pairs is in the scope of our future work. The creation of such array could also increase the SNR at larger regions compared to the common arrays with multitude of planar lateral coils. Such arrays are expected to find applications in spectroscopy and MRI.

## Methods

**Figure 2:**
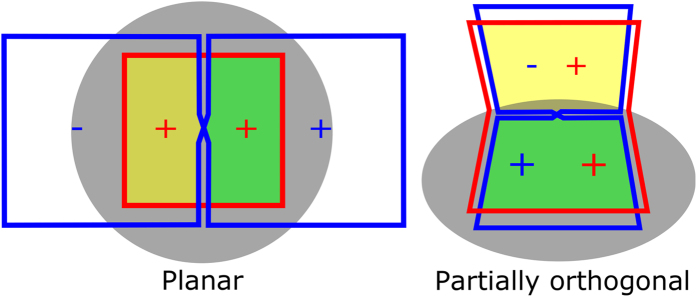
Schematic of a traditional planar concentric array in comparison to the proposed array. The planar array formed by a single loop (red) and butterfly coil (blue) contain an area of destructive interference of H-field (yellow) and the dimensions of the butterfly extend far beyond the area of interest (gray) for an optimal performance at the target depth. An array of a partially orthogonal single loop (red) and a partially orthogonal butterfly coil (blue). The orthogonal areas with opposing H-field directions (yellow areas) were used as active shielding in plane orthogonal to the plane with highest sensitivity to a sample (gray). The constructive interference (green areas) can be increased for enhanced **B**_1_ and more efficient use of planar physical space.

**Figure 3:**
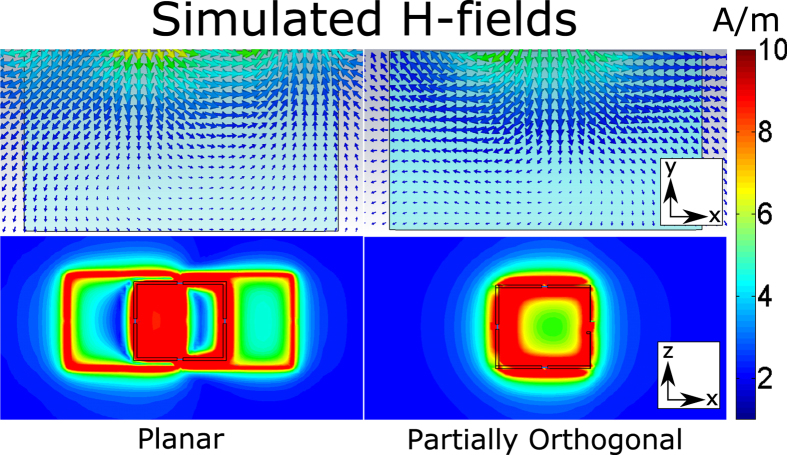
Reductions of H-field destructive interferences were found in the planar single loop and butterfly array in-phase. Using the proposed array of partially orthogonal coils, more directivity towards the scanned volume can be seen (vector plot, top) and the H-field destructive interferences were decreased as shown by an increase of H-field in the plane sensitive to the MRI signal (absolute plot, bottom).

**Figure 4:**
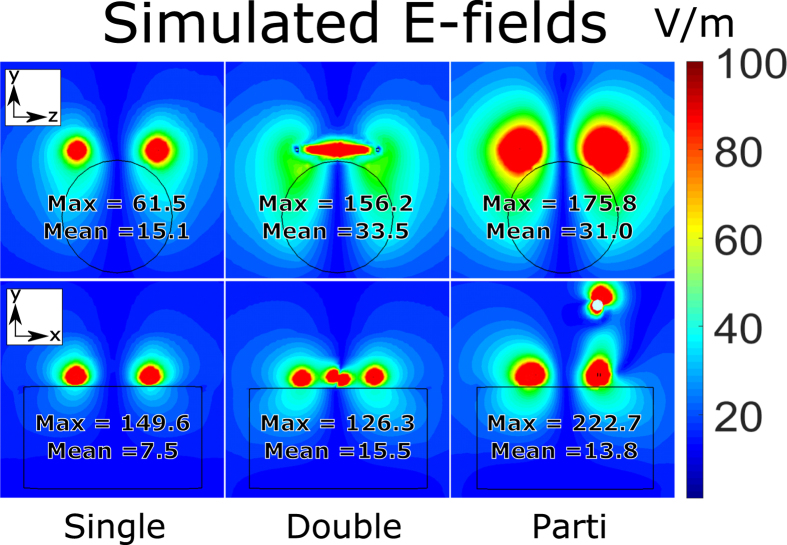
Simulated E-fields from the tested coil and arrays. Mean and maximum values were measured inside the phantom in the displayed slice. The partially orthogonal array (Parti) yielded higher maximum absolute E-field values which increase dielectric losses. However, mean values were reduced in comparison to the planar two-loop array (Double). This suggests mainly superficial losses.

**Figure 5:**
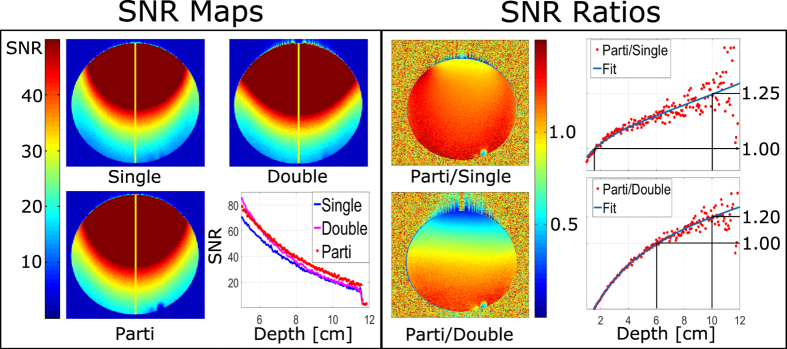
SNR maps and ratios acquired from phantom MRI measurements. Profile through the center of the images (yellow lines) corresponding to the single loop (Single), two loops with overlap (Double) and the partially orthogonal array (Parti) showed an increase of SNR at the target depth using the partially orthogonal array. The increase of SNR with increasing depth is also visible using a map of the SNR ratios (left). Profiles through the center were acquired and fitted with a bi-exponential fit to estimate the gain (right).

**Figure 6:**
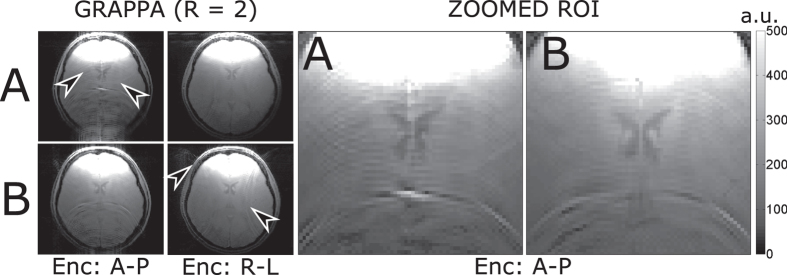
*In vivo* brain scans of a volunteer with GRAPPA as a parallel imaging data acquisition showed more pronounced artifacts when using the two single loops with overlap decoupling (**A**) in the anterior-posterior direction for phase encoding up to an approximately target distance of the coils. The artifacts present when using these planar coils (**A**) are shown with the arrows (**A**, Enc: A-P). Artifacts were more apparent when using the right-left direction used for phase encoding for the partially orthogonal coil array (**B**). The reduction in artifacts with higher signal using the proposed array (**B**) can be more clearly appreciated when zooming over a ROI.

All methods were carried out in accordance with relevant guidelines and regulations. An array of two partially orthogonal coils sensitive to virtually the same volume as shown in Fig. 2 was simulated, built and tested for a target depth of 10 cm at 3 T. The design was based on a single loop and a butterfly. The additional **B**_1_ field component generated by the butterfly due to the crossing of the wires dividing the lobes was pointed towards the sample for additional sensitivity. Common planar coil pairs were used for comparison in two steps. In the first step, we used electromagnetic numerical simulations to test the H-field interferences that arise when using concentric coils with the same phase and their impact on **B**_1_^−^. The partially orthogonal coil pair was simulated to show the reduction in destructive H-field interferences. In the second step, the proposed partially orthogonal array was compared to standard planar coil arrays. The standard coil arrays tested were the single loop as a reference and the two single loops with overlap decoupling. For the full comparison, the two standard planar arrays and the proposed partially orthogonal array were simulated, built and used to acquire MRI data. In the simulations, the coil arrays were compared in terms of decoupling and overall performance using a figure of merit at the target depth. In the measurements, SNR was evaluated from phantom datasets reconstructed using the maximum available combination algorithm^36^. Parallel imaging performance was evaluated using *in vivo* measurements.

### Simulations

Simulations were performed using CST Studio Suite 2016 (Computer Simulation Technology AG, Darmstadt, Germany). A time domain solver was employed. The coils were simulated using annealed copper with 3 mm thickness and 0.1 mm height. The first coil in each array was positioned 10 mm above a cylindrical phantom with 57.5 mm of radius and 200 mm of length. An electrical conductivity σ = 0.91 S/m and ε_r_ of 80 were assigned to the phantom whose length was oriented along the x-direction. Additional coils were separated by 3 mm in the y-direction. The performance of the single loop coil and the chosen coil arrays tested were evaluated using the absolute value of **B**_1_^−^ normalized to 1 Watt accepted power (|**B**_1_^−^|_norm_) as a figure of merit. In the case of the coil arrays, the scatter parameter (S21) was used to evaluate mutual coupling.

#### Simulated coils

A single loop with four splitting capacitors was simulated for reference. The single loop had optimized dimensions for the target depth i.e. inner length = 80 mm. The single loop was combined with a butterfly coil in-phase to illustrate H-field interferences and their impact in terms of **B**_1_^−^. The butterfly coil was modelled according to the optimized dimensions found in a previous work^37^ so each lobe had a length of 100 mm. Two single loop coils with overlap decoupling were simulated as well. The dimensions of the single loops were modified in order for the array to occupy approximately the same area as the single loop (80 × 79 mm^2^). The dimensions of the single loop coils were: inner length = 80 mm and inner width = 42 mm. Finally, the partially orthogonal coil pair proposed was simulated. The dimensions of the partially orthogonal coils covered approximately the same area as the planar single loop. However, the dimensions were modified to avoid positioning the copper tape directly on top of each other. This was made to facilitate their construction. The planar dimensions were for the single loop: 69.9 × 90 mm^2^ and for the butterfly: 80 × 80 mm^2^. The inner orthogonal areas were for the single loop: 76.9 × 90 mm^2^ (height x width) and for the butterfly: 80 × 80 mm^2^ with a space between the lobes of 1 mm.

For each coil and array, simulated E-fields in sagittal and transversal 2D slices going through the center of the phantom were exported to MATLAB (The MathWorks, Inc. Natick, USA). Mean and maximum values were found by selecting the area occupied by the phantom. FOVs including the coils and space surrounding the phantom were also plotted.

### Measurements

A 3 T MAGNETOM Trio a Tim MRI system (Siemens Healthcare AG, Erlangen, Germany) was used to perform the MRI measurements. The simulated, built and compared coils were: a single loop coil with the simulated dimensions, two loops with overlap decoupling, and the partially orthogonal coil pair. The coils were built using copper tape of 3 mm in width. The single loop and the two loops with overlap contained 4 splitting capacitors each to balance the current in the coils. 6 splitting capacitors were placed in each element of the partially orthogonal pair due to the total conductor length. The receiving chain in each coil included an active detuning circuit, a matching circuit and a cable trap before the pre-amplifier. After the pre-amplifier, a commercial cable system (Siemens Healthcare AG, Erlangen, Germany), with three cable traps was used to connect the coil arrays to the scanner. The scanner’s body coil was used for transmission. Two scans were acquired for the calculation of the SNR maps. The first scan used a transmission voltage automatically calculated by the scanner. The noise scan was obtained by setting the transmission voltage to 0 V.

#### Phantom

The setup for the MRI measurements included a cylindrical phantom with a diameter of 115 mm and length of 200 mm containing a solution of 3.75 g NiSO4 × 6 H2O + 5 g NaCL per 1000 g distilled water. The phantom was placed with its longest axis along the x-direction inside the bore. The tested coil and arrays were placed on a flat plastic surface with the phantom directly underneath. The distance between the phantom and the coil was fixed to avoid biasing.

Prior to MRI measurements, Q_Unloaded_ and Q_Loaded_ were measured with and without the phantom respectively for all individual elements. For this, the neighbor coil of the tested element in the two-coil arrays was opened at one point. The measurements were performed inductively via S12 measurements with a dual-loop probe^38^ using an E5063A ENA Series Network Analyzer (Keysight Technologies Deutschland GmbH, Boeblingen, Germany).

For the MRI measurements, a standard 2D spoiled gradient echo sequence was used to acquire the data using the following parameters: field-of-view = 143 × 143 mm, base resolution = 256, slice thickness = 5 mm, echo time = 10 ms, repetition time = 100 ms, flip angle = 90°, number of averages = 1, and bandwidth = 260 Hz/pixel.

#### In vivo

*In vivo* brain scans of one healthy volunteer were approved by the local Institutional Review Board (Medizinische Ethikkommission II of the Medical Faculty Mannheim, Heidelberg University) and performed with written informed consent. The *in vivo* measurements were acquired to show the performance of the arrays using the chosen parallel imaging technique. The setup included an ergonomic pillow to reduce unintended motion of the volunteer and a flat surface with fixed height where the coils were placed. The surface was used to maintain the distance between the coil and the volunteer’s head. Prior to MRI scanning, tuning and matching were performed with the same setup. The positioning of the coils inside the bore was replicated with the reference laser pointing at the center of the overlap in z-direction and the center of each coil in x-direction.

A standard 2D spoiled gradient echo was used with the slice position at the isocenter of the scanner. The parameters for the *in vivo* measurements were: field-of-view = 210 × 210 mm, base resolution = 128, slice thickness = 5 mm, echo time = 5 ms, repetition time = 2000 ms, flip angle = 90°, number of averages = 1 and bandwidth = 260 Hz/pixel. GRAPPA was used with a reduction factor of 2 as parallel imaging strategy.

## Additional Information

**How to cite this article**: Chacon-Caldera, J. *et al*. Partially orthogonal resonators for magnetic resonance imaging. *Sci. Rep.* **7**, 42347; doi: 10.1038/srep42347 (2017).

**Publisher's note:** Springer Nature remains neutral with regard to jurisdictional claims in published maps and institutional affiliations.

## Supplementary Material

Supplementary Information

## Figures and Tables

**Figure 1:**
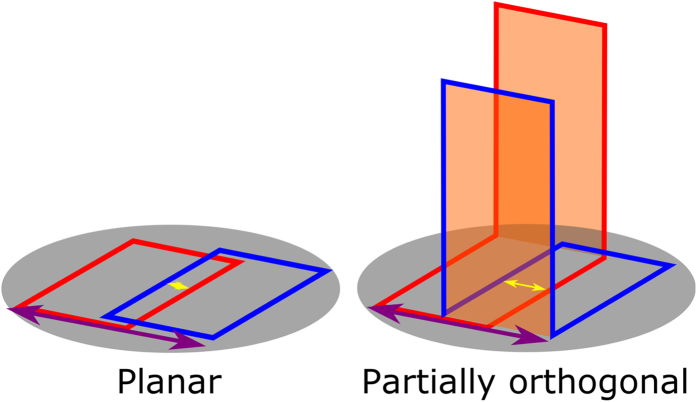
Planar and partially orthogonal arrays consisting of two single loops (red and blue) with overlap decoupling over the surface of a scanned volume (gray). The planar array requires a fixed overlap (yellow arrow) based on the dimensions of the loops which also determine the total area covered by the array (purple arrow). More flexibility in terms of overlap and total area covered by the array is added by using an orthogonal area (orange).

**Table 1:** Q factor measurements of the tested arrays.

	Single	Double (Loop 1, Loop 2)	Parti (Loop, Butterfly)
Q_Loaded_	26	50, 47	42, 39
Q_Unloaded_/Q_Loaded_	10.7	5.2, 5.4	4.1, 5.4
