# Supplementary material for: Partially orthogonal resonators for magnetic resonance imaging
Source: Sci Rep. 2017 Feb 10;7:42347. doi: 10.1038/srep42347 (PMC5301228; doi:10.1038/srep42347)
Supplement: Supplementary Information [file srep42347-s1.pdf]

Partially orthogonal resonators for magnetic resonance imaging

Jorge Chacon-Caldera*, Matthias Malzacher and Lothar R. Schad

Supplementary Information

The SNR in an MRI experiment for a single coil with a given protocol and measurement setup at a point r from the coil is given by:

$$SNR \propto \frac{\mathbf{B}_1(\mathrm{r})}{\sqrt{4kT\Delta f R_{eff}}}$$

Where k is the Boltzmann's constant, T is the temperature, Δf is the bandwidth of the measurement and the R_{eff} is the effective resistance given by the sample, the coil and the electronics as in equation 1. In the case of arrays of coils, $\mathbf{B}_1(\mathrm{r})$ is given by a weighted sum of the individual coils. The resistances are added linearly with an additional component of shared noise between the coils which can be found using the noise correlations between channels and accounted during the coil combination.

The noise sources per channel can be further compressed into R_{sample} and R_{coil} where the noise from the electronics can be included in R_{coil}. Having these two sources of noise, the dominance can be found using the ratio of Q-factors of the coil in the unloaded and loaded case found using:

$$Q_{unloaded} = \frac{\omega L}{R_{coil}}$$

$$Q_{loaded} = \frac{\omega L}{R_{coil} + R_{sample}}$$

Where $Q_{unloaded}/Q_{loaded} = 2$ indicates equal noise contribution from the noise and the sample, greater ratios indicate dominance of the sample noise [9].
